# A Role for Auxin in Ethylene-Dependent Inducible Aerenchyma Formation in Rice Roots

**DOI:** 10.3390/plants9050610

**Published:** 2020-05-11

**Authors:** Takaki Yamauchi, Akihiro Tanaka, Nobuhiro Tsutsumi, Yoshiaki Inukai, Mikio Nakazono

**Affiliations:** 1 Japan Science and Technology Agency, PRESTO, Kawaguchi, Saitama 332-0012, Japan; 2 Graduate School of Agricultural and Life Sciences, The University of Tokyo, Bunkyo, Tokyo 113-8657, Japan; atsutsu@mail.ecc.u-tokyo.ac.jp; 3 Graduate School of Bioagricultural Sciences, Nagoya University, Nagoya, Aichi 464–8601, Japan; tanaka.akihiro.j@outlook.com; 4 International Center for Research and Education in Agriculture, Nagoya University, Nagoya, Aichi 464–8601, Japan; inukaiy@agr.nagoya-u.ac.jp; 5 The UWA School of Agriculture and Environment, Faculty of Science, The University of Western Australia, Crawley, WA 6009, Australia

**Keywords:** aerenchyma, auxin, ethylene, rice (*Oryza sativa*), root, waterlogging

## Abstract

Internal oxygen diffusion from shoot to root tips is enhanced by the formation of aerenchyma (gas space) in waterlogged soils. Lysigenous aerenchyma is created by programmed cell death and subsequent lysis of the root cortical cells. Rice (*Oryza sativa*) forms aerenchyma constitutively under aerobic conditions and increases its formation under oxygen-deficient conditions. Recently, we have demonstrated that constitutive aerenchyma formation is regulated by auxin signaling mediated by Auxin/indole-3-acetic acid protein (AUX/IAA; IAA). While ethylene is involved in inducible aerenchyma formation, the relationship of auxin and ethylene during aerenchyma formation remains unclear. Here, we examined the effects of oxygen deficiency and ethylene on aerenchyma formation in the roots of a rice mutant (*iaa13*) in which auxin signaling is suppressed by a mutation in the degradation domain of IAA13 protein. The results showed that AUX/IAA-mediated auxin signaling contributes to ethylene-dependent inducible aerenchyma formation in rice roots. An auxin transport inhibitor abolished aerenchyma formation under oxygen-deficient conditions and reduced the expression of genes encoding ethylene biosynthesis enzymes, further supporting the idea that auxin is involved in ethylene-dependent inducible aerenchyma formation. Based on these studies, we propose a mechanism that underlies the relationship between auxin and ethylene during inducible aerenchyma formation in rice roots.

## 1. Introduction

Internal oxygen movement from shoot to roots through aerenchyma is essential for plants to adapt to waterlogged soils [1]. Lysigenous aerenchyma in roots is created by programmed cell death (PCD) and subsequent lysis of the cortical cells [2,3]. In roots of upland plants, such as maize (*Zea mays* ssp. *mays*) and wheat (*Triticum aestivum*), lysigenous aerenchyma is not generally formed under aerobic conditions, but its formation is induced under oxygen-deficient conditions [4,5,6,7]. In roots of the wetland plant rice (*Oryza sativa*), lysigenous aerenchyma is formed constitutively even under aerobic conditions (constitutive aerenchyma formation), and its formation is further increased under oxygen-deficient conditions (inducible aerenchyma formation) [4].

Ethylene is involved in inducible aerenchyma formation in rice roots [4,5,6,7]. Under waterlogging, lower diffusion rates of gases to the rhizosphere enhance the ethylene accumulation in roots [8,9]. Ethylene is biosynthesized by the conversion of S-adenosylmethionine to 1-amino-cyclopropane-1-carboxylic acid (ACC) by ACC synthase (ACS) and that of ACC to ethylene by ACC oxidase (ACO) [10]. During inducible aerenchyma formation in rice roots, the expression levels of *ACS1* and *ACO5* are increased, and they contribute to increased ethylene content in the roots [11]. Moreover, ethylene-induced production of reactive oxygen species (ROS) by respiratory burst oxidase homolog H (RBOHH) is involved in inducible aerenchyma formation in rice roots [12]. Ethylene and ROS signaling is also involved in lysigenous aerenchyma formation in rice shoots [13].

Auxin signaling is mediated by a family of transcription factors called auxin response factors (ARFs) [14]. ARF-dependent transcriptional regulation is repressed by the binding of auxin/indole-3-acetic acid proteins (AUX/IAAs; IAAs) to ARFs [15]. The rice genome has 25 *ARF* genes and 31 *IAA* genes [16,17]. IAA proteins have a conserved amino acid sequence motif (AUX/IAA domain II), which is required for auxin-dependent proteolysis of the IAA proteins [18]. The auxin signaling is suppressed in roots of the gain of function (dominant-negative) *iaa13* mutant having a single amino acid substitution in the AUX/IAA domain II of IAA13 protein [19]. Although until recently it remained unclear what triggers constitutive aerenchyma formation in rice roots [6], we demonstrated that constitutive aerenchyma formation in rice roots is regulated by the auxin signaling through the functional analysis of the *iaa13* mutant [20].

Exogenous treatment with ethylene stimulates aerenchyma formation in rice roots even under aerobic conditions [21,22]. While inhibitors of ethylene perception or ethylene action reduce aerenchyma formation in rice roots under oxygen-deficient conditions [12,21], they cannot abolish aerenchyma formation under either oxygen-deficient or aerobic conditions [12,21,23]. On the other hand, an auxin transport inhibitor completely blocks constitutive aerenchyma formation in rice roots under aerobic conditions [20], implying that auxin signaling is required for the ethylene-dependent aerenchyma formation. However, the relationship between auxin and ethylene during inducible aerenchyma formation remains unclear.

The objective of this study was to test the possibility that auxin is involved in ethylene-dependent aerenchyma formation. To this end, we used the *iaa13* mutant in which the dominant negative IAA13 suppressed auxin signaling in the roots [19]. We examined the effect of enhancing ethylene signaling on aerenchyma formation in roots of *iaa13* and its wild type (WT; cv. Taichung 65; T65). We also examined the effects of an auxin transport inhibitor on ethylene-dependent aerenchyma formation and the expression levels of genes encoding ethylene biosynthesis enzymes in the roots of the WT. Finally, we examined the effects of an ethylene precursor on aerenchyma formation in the presence of the auxin transport inhibitor. Our results strongly suggest that auxin is involved in the regulation of ethylene-dependent inducible aerenchyma formation in rice roots.

## 2. Results

### 2.1. Effect of Oxygen Deficiency on Aerenchyma Formation

**Figure 1:**
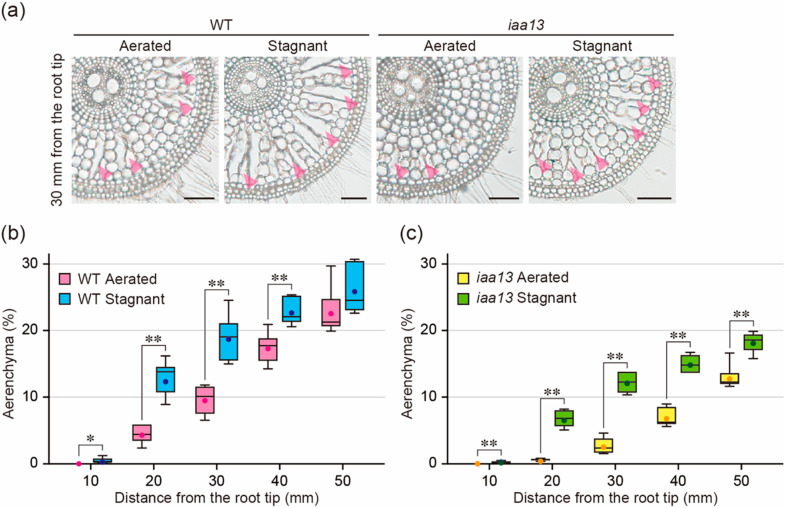
Aerenchyma formation under aerated or stagnant conditions. (**a**) Cross-sections at 30 mm from the tips of adventitious roots of the wild-type (WT) and *iaa13* mutant. Aerenchyma is indicated by magenta arrowheads. Bars = 100 μm. (**b**,**c**) Percentages of aerenchyma in root cross-sectional area at 10, 20, 30, 40, and 50 mm. Twenty-day-old aerobically grown WT (**b**) and *iaa13* (**c**) seedlings were further grown under aerated or stagnant conditions for 48 h. (**b**,**c**) Significant differences between the conditions at *p* < 0.01 are denoted by ** (two-sample *t*-test). Boxplots show the median (horizontal lines), 25th to 75th percentiles (edges of the boxes), minimum to maximum (edges of the whiskers), and mean values (dots in the boxes) (*n* = 6).

During inducible aerenchyma formation under oxygen-deficient conditions, ethylene accumulation increases in rice roots [11,12]. To test the effect of oxygen deficiency on aerenchyma formation in *iaa13*, 20-d-old aerobically grown WT and *iaa13* seedlings were transferred to aerated or stagnant (deoxygenated) conditions, which mimic the changes in gas composition in waterlogged soils [24], for 48 h. After 48 h, root elongation of the WT was 12.9% less under stagnant conditions than under aerated conditions, while root elongation of *iaa13* was 18.2% less under stagnant conditions (Supplemental Figure S1a). Subsequently, transverse sections along the adventitious roots were prepared (Figure 1a), and the percentage of each cross-section occupied by aerenchyma was determined (Figure 1b,c). Aerenchyma formation in the WT roots was significantly higher at 10, 20, 30, and 40 mm under stagnant conditions than under aerated conditions (Figure 1a,b), whereas aerenchyma formation in the *iaa13* roots was significantly higher at all positions under stagnant conditions (Figure 1a,c). Aerenchyma formation in the WT at 20, 30, 40, and 50 mm was significantly higher than that in *iaa13* both under aerated and stagnant conditions (Supplemental Figure S2a,b), suggesting that difference in aerenchyma formation between the WT and *iaa13* under stagnant conditions is largely affected by the reduced constitutive aerenchyma formation in the *iaa13* roots.

### 2.2. Effect of an Ethylene Precursor on Aerenchyma Formation

**Figure 2:**
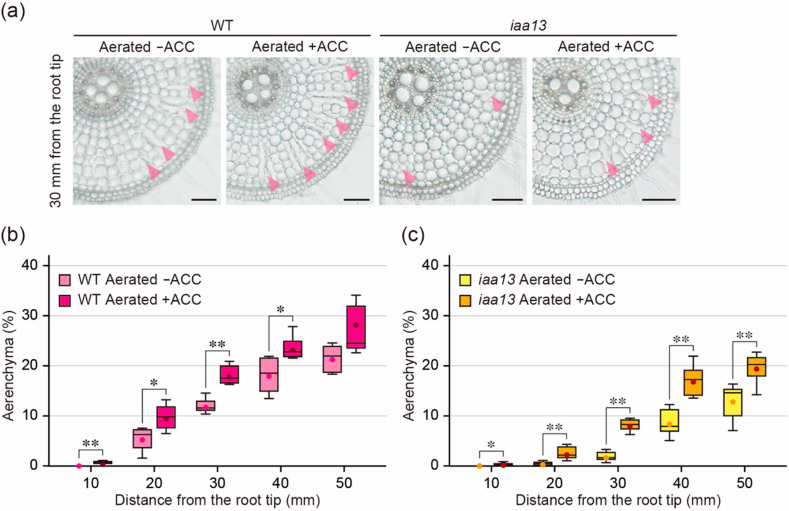
Aerenchyma formation under aerated conditions with or without 1-aminocyclopropane-1-carboxylic acid (ACC) treatment. (**a**) Cross-sections at 30 mm from the tips of adventitious roots of the wild type (WT) and *iaa13* mutant. Aerenchyma is indicated by magenta arrowheads. Bars = 100 μm. (**b**,**c**) Percentages of aerenchyma in root cross-sectional area at 10, 20, 30, 40, and 50 mm. Twenty-day-old aerobically grown WT (**b**) and *iaa13* (**c**) seedlings were further grown under aerated conditions with or without 10 µM ACC for 48 h. (**b**,**c**) Significant differences between the conditions at *p* < 0.05 and *p* < 0.01 are denoted by * and **, respectively (two-sample *t*-test). Boxplots show the median (horizontal lines), 25th to 75th percentiles (edges of the boxes), minimum to maximum (edges of the whiskers) and mean values (dots in the boxes) (*n* = 6).

Exogenously supplied ethylene stimulates aerenchyma formation in rice roots even under aerobic conditions [21,22], and the treatment with an ethylene precursor ACC also induces its formation [11,23]. To further investigate the effect of ethylene on aerenchyma formation in the *iaa13* roots, 20-d-old aerobically grown WT and *iaa13* seedlings were transferred to aerated conditions with or without 10 µM ACC. After 48 h, root elongation of the WT was 15.1% less under aerated conditions with ACC than without ACC, while root elongation of *iaa13* was 18.7% less under aerated conditions with ACC (Supplemental Figure S1b). The suppression of root elongation in both the WT and *iaa13* by ACC treatment (Supplemental Figure S1b) was similar to the suppression of root elongation in both the WT and *iaa13* by stagnant conditions (Supplemental Figure S1a). This suggests that ethylene accumulation, which is stimulated by ACC or oxygen deficiency, has similar effects on root elongation in the WT and *iaa13*. As is the case with stagnant conditions, aerenchyma formation in the WT roots was significantly higher at 10, 20, 30, and 40 mm from the root tips under aerated conditions with ACC than without ACC (Figure 2a,b), whereas aerenchyma formation in the *iaa13* roots was significantly higher at all positions under aerated conditions with ACC (Figure 2a,c). Aerenchyma formation in the WT at 20, 30, 40 and 50 mm was significantly higher than that in *iaa13* under aerated conditions without ACC, and aerenchyma formation in the WT at 20, 30, and 40 mm was significantly higher than that in *iaa13* under aerated conditions with ACC (Supplemental Figure S2c,d).

### 2.3. Differences in Response to Oxygen Deficiency and ACC between the Wild Type and iaa13

**Figure 3:**
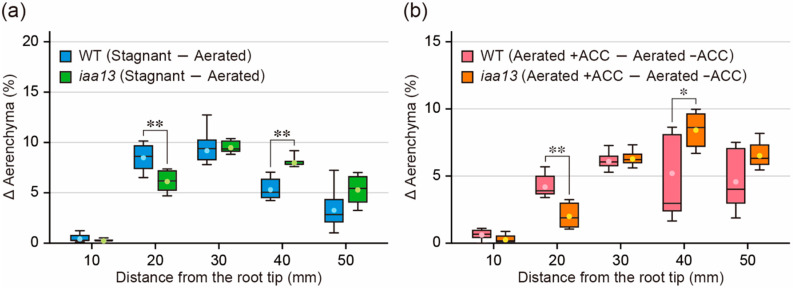
Differences in aerenchyma formation between under aerated and stagnant conditions and between aerated conditions with and without 1-aminocyclopropane-1-carboxylic acid (ACC) treatment. (**a**,**b**) Differences in the percentages of aerenchyma (D Aerenchyma (%)) in root cross-sectional area at 10, 20, 30, 40, and 50 mm from the tips of adventitious roots of the wild type (WT) and *iaa13* mutant. The differences in the percentages of aerenchyma were calculated by the data obtained in Figure 1a and Figure 2b, respectively. (**a**,**b**) Significant differences between the genotypes at *p* < 0.05 and *p* < 0.01 are denoted by * and **, respectively (two-sample *t*-test). Boxplots show the median (horizontal lines), 25th to 75th percentiles (edges of the boxes), minimum to maximum (edges of the whiskers) and mean values (dots in the boxes) (*n* = 6).

Both oxygen deficiency (Figure 1c) and ACC (Figure 2c) increased aerenchyma formation in *iaa13* roots, suggesting that ethylene-dependent inducible aerenchyma formation is not reduced in *iaa13*. Interestingly, longitudinal patterns of the differences in aerenchyma formation between aerated and stagnant conditions (Figure 3a), and between aerated conditions with and without ACC (Figure 3b), were similar to each other. At 10 to 20 mm from the root tips, the differences were larger in the WT roots than in *iaa13* (Figure 3a,b). The differences were comparable at 30 mm and then became smaller in the WT than those in *iaa13* at 40 to 50 mm (Figure 3a,b). These results suggest that ethylene-dependent aerenchyma formation in *iaa13* is reduced at the apical part of the roots.

### 2.4. Effect of an Auxin Transport Inhibitor on Aerenchyma Formation

**Figure 4:**
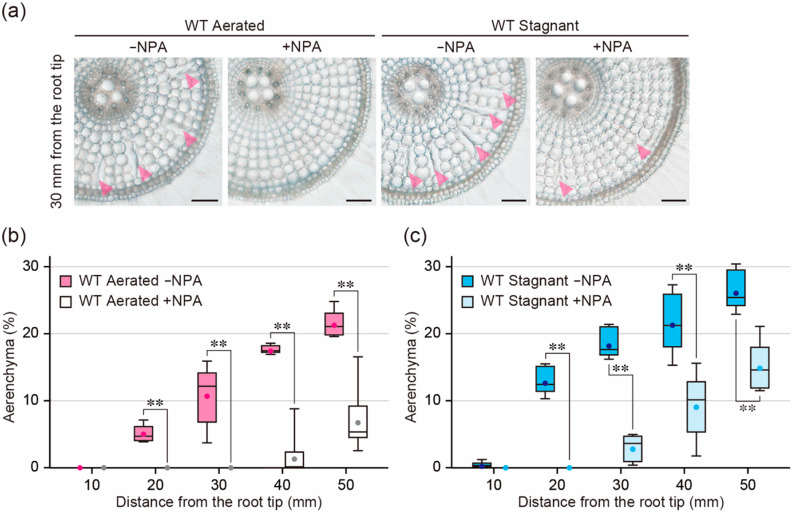
Aerenchyma formation under aerated or stagnant conditions with or without *N*-1-naphthylphthalamic acid (NPA) treatment. (**a**) Cross-sections at 30 mm from the tips of adventitious roots of the wild type (WT). Aerenchyma is indicated by magenta arrowheads. Bars = 100 μm. (**b**,**c**) Percentages of aerenchyma in root cross-sectional area at 10, 20, 30, 40, and 50 mm. Twenty-day-old aerobically grown WT seedlings were further grown under aerated (**b**) or stagnant (**c**) conditions with or without 0.5 µM NPA for 48 h. (**b**,**c**) Significant differences between the conditions at *p* < 0.01 are denoted by ** (two-sample *t*-test). Boxplots show the median (horizontal lines), 25th to 75th percentiles (edges of the boxes), minimum to maximum (edges of the whiskers) and mean values (dots in the boxes) (*n* = 6).

To confirm the effect of auxin on the ethylene-dependent aerenchyma formation, we examined the effect of the auxin transport inhibitor *N*-1-naphthylphthalamic acid (NPA) on aerenchyma formation. Twenty-day-old aerobically grown WT seedlings were transferred to aerated or stagnant conditions with or without 0.5 µM NPA. After 48 h, root elongation of the WT was 19.0% less under aerated conditions with NPA than without NPA, while root elongation of it was 17.4% less under stagnant conditions with NPA (Supplemental Figure S1c). Root elongation in 48 h NPA treatment was 31.3 ± 3.4 mm under aerated conditions and 26.8 ± 4.7 mm under stagnant conditions (Supplemental Figure S1c). These results indicate that root cortical cells at 10 to 30 mm under aerated conditions and at 10 to 20 mm under stagnant conditions are generated in the presence of NPA. Under aerated conditions, NPA completely blocked aerenchyma formation at 10 to 30 mm from the root tips (Figure 4a,b). Interestingly, NPA also completely blocked aerenchyma formation at 10 to 20 mm under stagnant conditions (Figure 4a,c). These results strongly suggest that auxin is also involved in ethylene-dependent aerenchyma formation in rice roots.

### 2.5. Effect of NPA on the Expression of Ethylene Biosynthesis Genes

**Figure 5:**
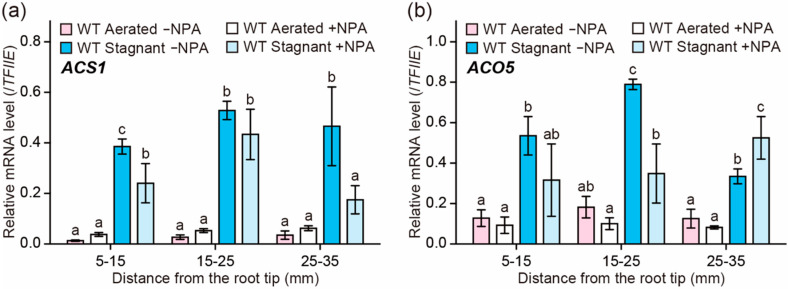
Expression of ethylene biosynthesis genes in adventitious roots under aerated or stagnant conditions with or without *N*-1-naphthylphthalamic acid (NPA) treatment. Twenty-day-old aerobically grown wild type (WT) seedlings were further grown under aerated or stagnant conditions with or without 0.5 µM NPA for 48 h. Relative transcription levels of *ACS1* (**a**) and *ACO5* (**b**) at 5–15, 15–25, and 25–35 mm from the tips of adventitious roots. The gene encoding transcription initiation factor IIE (TFIIE) was used as a control. Different lowercase letters denote significant differences among the conditions (*p* < 0.05, one-way ANOVA followed by Tukey’s test for multiple comparisons). Values are means ± SD (*n* = 3).

To test the effect of the auxin transport inhibitor on the ethylene biosynthesis in rice roots, the transcript levels of ethylene biosynthesis genes were analyzed by quantitative reverse transcription (qRT)-PCR analysis. Previously, we showed that, among six *ACS* and seven *ACO* genes in the rice genome, *ACS1* and *ACO5* had the highest transcript levels during inducible aerenchyma formation in rice roots [23]. The transcript levels of *ACS1* and *ACO5* were significantly increased under stagnant conditions and peaked at 15–25 mm from the root tips (Figure 5a,b), where aerenchyma formation is highly induced (at 20 mm; Figure 4c). NPA significantly reduced the transcript level of *ACS1* at 5–15 mm and 25–35 mm (Figure 5a), and it also reduced that of *ACO5* at 15–25 mm (Figure 5b). By contrast, under aerated conditions, the transcript levels of *ACS1* and *ACO5* with NPA treatment were comparable to those without NPA treatment (Figure 5a,b). These results suggest that auxin contributes to the transcriptional induction of the *ACS1* and *ACO5* genes under stagnant conditions.

### 2.6. Effect of ACC on Aerenchyma Formation in the Presence of NPA

**Figure 6:**
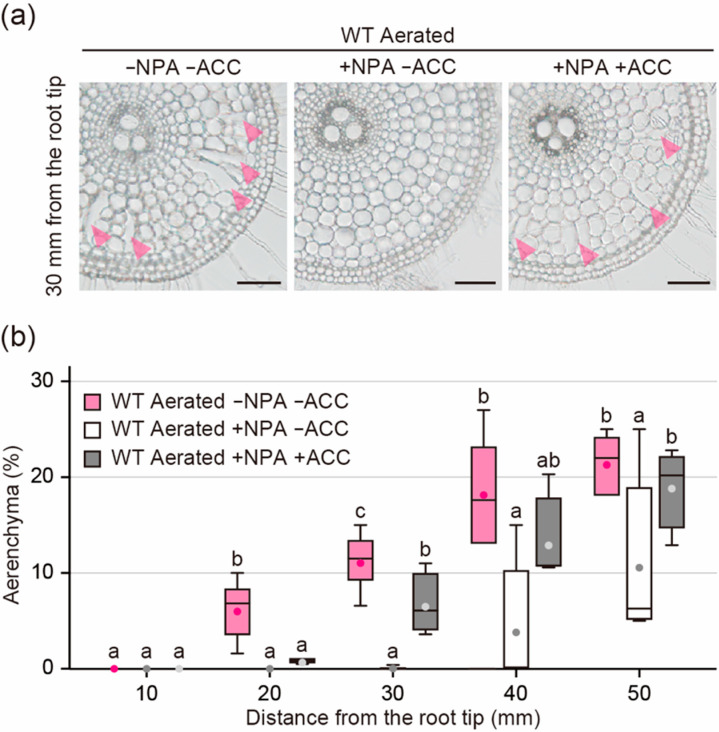
Aerenchyma formation under aerated conditions with or without *N*-1-naphthylphthalamic acid (NPA) or 1-aminocyclopropane-1-carboxylic acid (ACC) treatments. (**a**) Cross-sections at 30 mm from the tips of adventitious roots of the wild type (WT). Aerenchyma is indicated by magenta arrowheads. Bars = 100 μm. (**b**) Percentages of aerenchyma in root cross-sectional area at 10, 20, 30, 40, and 50 mm. Twenty-day-old aerobically grown WT seedlings were further grown under aerated conditions without NPA and ACC, with 0.5 µM NPA and without ACC or with 0.5 µM NPA and 10 µM ACC for 48 h. Different lowercase letters denote significant differences among the conditions (*p* < 0.05, one-way ANOVA followed by Tukey’s test for multiple comparisons). Boxplots show the median (horizontal lines), 25th to 75th percentiles (edges of the boxes), minimum to maximum (edges of the whiskers), and mean values (dots in the boxes) (*n* = 4–6).

The reduction of ACS1 and ACO5 genes in the WT roots by the NPA treatment suggested that auxin stimulates ethylene biosynthesis through the transcriptional regulation of ethylene biosynthesis genes. To test this hypothesis, the WT roots were treated with the ethylene precursor ACC in the presence of NPA. Twenty-day-old aerobically grown WT seedlings were transferred to aerated conditions with or without 0.5 µM NPA and/or 10 µM ACC. After 48 h, root elongation of NPA treated seedlings (31.3 ± 3.4 mm) was 25.4% less than that of untreated seedlings (42.0 ± 7.0 mm), while root elongation of NPA- and ACC-treated seedlings (14.8 ± 4.1 mm) was 64.9% less than that of untreated seedlings (Supplemental Figure S1d). NPA almost completely blocked aerenchyma formation at 10 to 30 mm from the root tips (Figure 6a,b), whereas ACC restored its formation at 30, 40, and 50 mm (Figure 6a,b). These results indicate that the prevention of ethylene-dependent aerenchyma formation by NPA is at least partly canceled by adding the ethylene precursor ACC.

## 3. Discussion

The present results demonstrate that auxin is required for inducible aerenchyma formation in rice roots. Aerenchyma formation in the WT and *iaa13* roots was induced by the growth under stagnant conditions (Figure 1b,c), and under aerated conditions with an ethylene precursor ACC treatment (Figure 2b,c). These results suggest that oxygen deficiency and ethylene can stimulate inducible aerenchyma formation in *iaa13*. On the other hand, the mutated *IAA13* (*iaa13*) gene is expressed predominantly at the apical part of the *iaa13* roots, and the dominant negative effect of the mutated IAA13 protein on aerenchyma formation is restricted to the apical part of the roots [20]. Moreover, the curly root phenotype in the rice *pin2* mutant, which is caused by the asymmetric auxin distribution at the apical part of the roots, is rescued in roots of the *pin2 iaa13* double mutant [25]. These observations suggest that the mutated IAA13 protein affects ethylene-dependent aerenchyma formation at the apical part of the roots. Indeed, the difference in the levels of aerenchyma formation at 20 mm from the root tips between aerated and stagnant conditions, and between aerated conditions with and without ACC, was significantly lower in *iaa13* than that in the WT (Figure 3a,b). These results suggest that the AUX/IAA-mediated auxin signaling is involved in ethylene-dependent inducible aerenchyma formation under oxygen-deficient conditions.

During constitutive aerenchyma formation in rice roots, the auxin transport inhibitor NPA completely blocks the death of the cortical cells [20]. NPA also abolished aerenchyma formation at 10 to 20 mm from the root tips under stagnant conditions (Figure 4c). As the elongation of the WT roots with NPA was ~25 mm under stagnant conditions (Supplemental Figure S1c), aerenchyma formation in the cortex was completely blocked by NPA even under stagnant conditions. These results are interesting because they showed that auxin is required not only for constitutive aerenchyma formation but also for inducible aerenchyma formation in rice roots. In Arabidopsis roots, exogenous ACC treatment was found to reduce lateral root formation by enhancing auxin level at the apical part of the roots, whereas a knockout mutant of the ethylene-signaling gene *ETHYLENE INSENSITIVE2* (*EIN2*) is defective in these responses [26]. Similar observations were reported in the apical hook formation [27] and the control of root gravitropism [28], all of which are directly regulated by the auxin signaling [29,30,31]. If this is also the case for aerenchyma formation, it is reasonable that inhibition of auxin transport from the shoots to root tips by NPA abolished aerenchyma formation under oxygen-deficient conditions (Figure 4c).

On the other hand, another possibility is that auxin affects ethylene biosynthesis during inducible aerenchyma formation in rice roots, as the NPA treatment reduced the expression levels of *ACS1* and *ACO5* (which have highest expression levels among the *ACS* and *ACO* homologs in rice roots during inducible aerenchyma formation [23]) under stagnant conditions (Figure 5a,b). In Arabidopsis, some *ACS* and *ACO* genes are transcriptionally activated by auxin [32,33,34]. In roots of maize, exogenous natural auxin treatment increases aerenchyma formation, possibly by stimulating ethylene biosynthesis [35]. In maize roots, auxin-dependent constitutive aerenchyma formation does not generally occur under aerobic conditions [36,37,38], which suggests that exogenous auxin stimulates the ethylene-dependent pathway [35]. These results further support the idea that auxin is involved in ethylene-dependent aerenchyma formation through the control of ethylene biosynthesis. Indeed, the exogenous treatment of ACC partly restored aerenchyma formation in the WT roots in the presence of NPA (Figure 6b). Although the expression level of *ACO5* was decreased by the NPA treatment (Figure 5b), the exogenously supplied ACC could still enhance the ethylene production by the remaining activity of ACOs, thereby stimulating ethylene-dependent inducible aerenchyma formation in the WT roots (Figure 6b). Similar observations were previously obtained for a rice mutant, which has lower expression levels of *ACS1* and *ACO5* in the roots [11,23]. So far, we cannot rule out the possibility that the reduced root elongation rate by NPA and ACC affects the amounts of aerenchyma formation, as the root elongation of the WT is severely reduced by the NPA and ACC treatments (Supplemental Figure S1d). Further studies using the auxin and ethylene biosynthesis and/or signaling mutants are needed to understand the molecular mechanisms underlying the relationship between auxin and ethylene during inducible aerenchyma formation and how this relationship contributes to the fine-tuning of lysigenous aerenchyma formation in rice roots under oxygen-deficient conditions.

## 4. Materials and Methods

### 4.1. Plant Materials and Growth Conditions

Seeds of the rice *iaa13* mutant [19] and its background wild type (cv. Taichung 65; T65) were sterilized in 0.5% (*v*/*v*) sodium hypochlorite for 30 min and rinsed with deionized water. The seeds were germinated on petri dishes filled up with deionized water and put in a growth chamber at 28 °C under dark conditions. After 2 days, seeds were placed on a mesh floating on top of an aerated quarter strength nutrient solution at 28 °C under 24 h light conditions (photosynthetically active radiation, 200–250 μmol m^−2^ s^−1^) for 4 d. The composition of the nutrient solution is described by Colmer et al. [22]. Seedlings (6-d-old) were then transferred to 5-L pots (8–12 plants per pot, 250 mm height × 120 mm length × 180 mm width) containing aerated full-strength nutrient solution. After 7 days, 13-day-old rice plants were transferred newly prepared full-strength nutrient solution and further grown for 7 days. Twenty-day-old seedlings were transferred to 5-L pots containing an aerated full-strength nutrient solution or stagnant solution. Stagnant solution, which mimics waterlogged soils [24], contained 0.1% (*w*/*v*) dissolved agar and was deoxygenated (dissolved O_2_, <0.5 mg L^−1^) prior to use by flushing with N_2_ gas.

### 4.2. Chemical Treatments

For each treatment, 20-day-old rice seedlings were transferred to 2-L pots (4 plants per pot, 250 mm height × 80 mm length × 120 mm width) containing nutrient solution. For the 1-aminocyclopropane-1-carboxylic acid (ACC) and *N*-1-naphthylphthalamic acid (NPA) treatments, 20-d-old aerobically grown rice seedlings were further grown in aerated or stagnant nutrient solutions with or without 10 µM ACC and/or with or without 0.5 µM NPA for 48 h. The stock solutions of ACC and NPA (both Sigma-Aldrich) were prepared to 100 mM in sterilized water and dimethylformamide, respectively.

### 4.3. Anatomical Observations

Root cross-sections were prepared from 4-mm-long segments of adventitious roots. For analysis of aerenchyma formation, root segments were cut at the indicated distances (±2 mm) from the tips of adventitious roots. Cross-sections were prepared by hand sectioning with a razor blade. The root cross-sections were photographed using an optical microscope (BX60; OLYMPUS) with a charge-coupled device (CCD) camera (DP70; OLYMPUS). The percentages of each cross-section occupied by aerenchyma were determined with ImageJ software (Ver. 1.43u, US National Institutes of Health).

### 4.4. qRT-PCR Analysis

Root segments at the indicated distances from the tips of adventitious roots were ground in liquid nitrogen. Total RNA was extracted from the frozen fixed tissues using a RNeasy Plant Mini Kit (QIAGEN) according to the instructions of the manufacturer. Transcript levels were measured using a StepOnePlus Real-Time PCR System (Applied Biosystems) and One Step SYBR PrimeScript RT-PCR Kit II (Takara Bio) as described by Yamauchi et al. [12]. Transcript levels were normalized to the transcript level of *transcription initiation factor IIE* (*TFIIE*). Primer sequences used for the qRT-PCR are shown in Supplemental Table S1.

### 4.5. Statistical Analyses

Statistical differences between means were calculated using two-sample *t*-tests. For multiple comparisons, data were analyzed by one-way ANOVA and post hoc Tukey’s test using SPSS Statistics Version 25 (IBM Software).

## Supplementary Materials

The following are available online at https://www.mdpi.com/2223-7747/9/5/610/s1, Figure S1: Root elongation under aerated or stagnant conditions with or without the inhibitor treatments; Figure S2: Aerenchyma formation under aerated or stagnant conditions or aerated conditions with or without 1-aminocyclopropane-1-carboxylic acid (ACC) treatment; Table S1: List of primers used for qRT–PCR analysis.
