# Supplementary material for: A Role for Auxin in Ethylene-Dependent Inducible Aerenchyma Formation in Rice Roots
Source: Plants (Basel). 2020 May 11;9(5):610. doi: 10.3390/plants9050610 (PMC7284992; doi:10.3390/plants9050610)
Supplement: Supplementary file 1 [file plants-09-00610-s001.pdf]

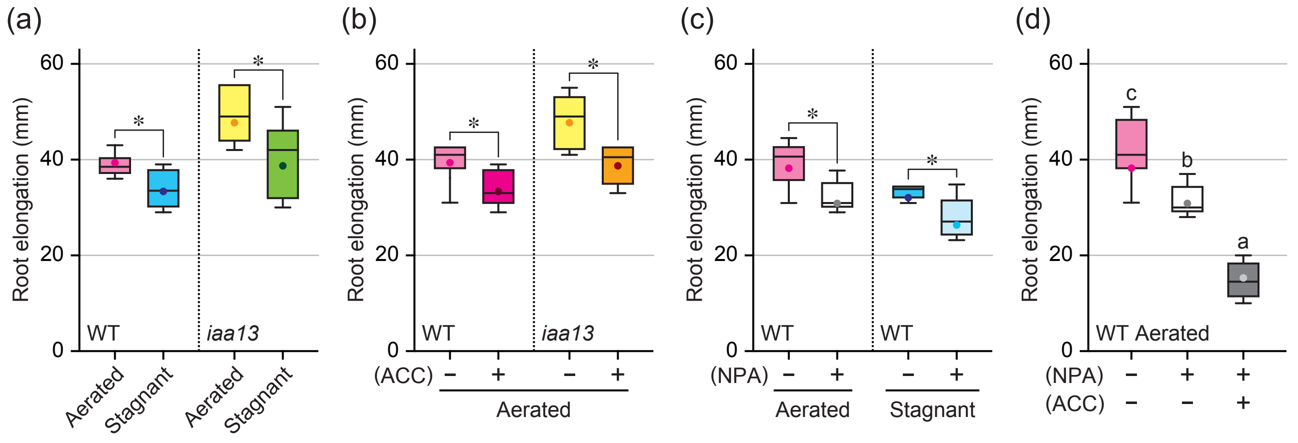

Supplemental Figure S1. Root elongation under aerated or stagnant conditions with or without inhibitor treatments. (**a**) Elongation of adventitious roots of the wild type (WT) and *iaa13* mutant in 48 h under aerated or stagnant conditions. (**b**) Elongation of the WT and *iaa13* roots in 48 h under aerated conditions with or without 10 μM 1-aminocyclopropane-1-carboxylic acid (ACC) treatment. (**c**) Elongation of the WT roots in 48 h under aerated or stagnant conditions with or without 0.5 μM *N*-1-naphthylphthalamic acid (NPA) treatment. (**d**) Elongation of the WT roots in 48 h under aerated conditions with or without 0.5 μM NPA or 10 μM ACC treatments. (**a-c**) Significant differences between the conditions at $P < 0.05$ are denoted by * (two-sample *t* test). (**d**) Different lowercase letters denote significant differences among the conditions ($P < 0.05$, one-way ANOVA followed by Tukey' s test for multiple comparisons). Boxplots show the median (horizontal lines), 25th to 75th percentiles (edges of the boxes), minimum to maximum (edges of the whiskers) and mean values (dots in the boxes) [n = 6 (**a-c**), n = 4-6 (**d**)].

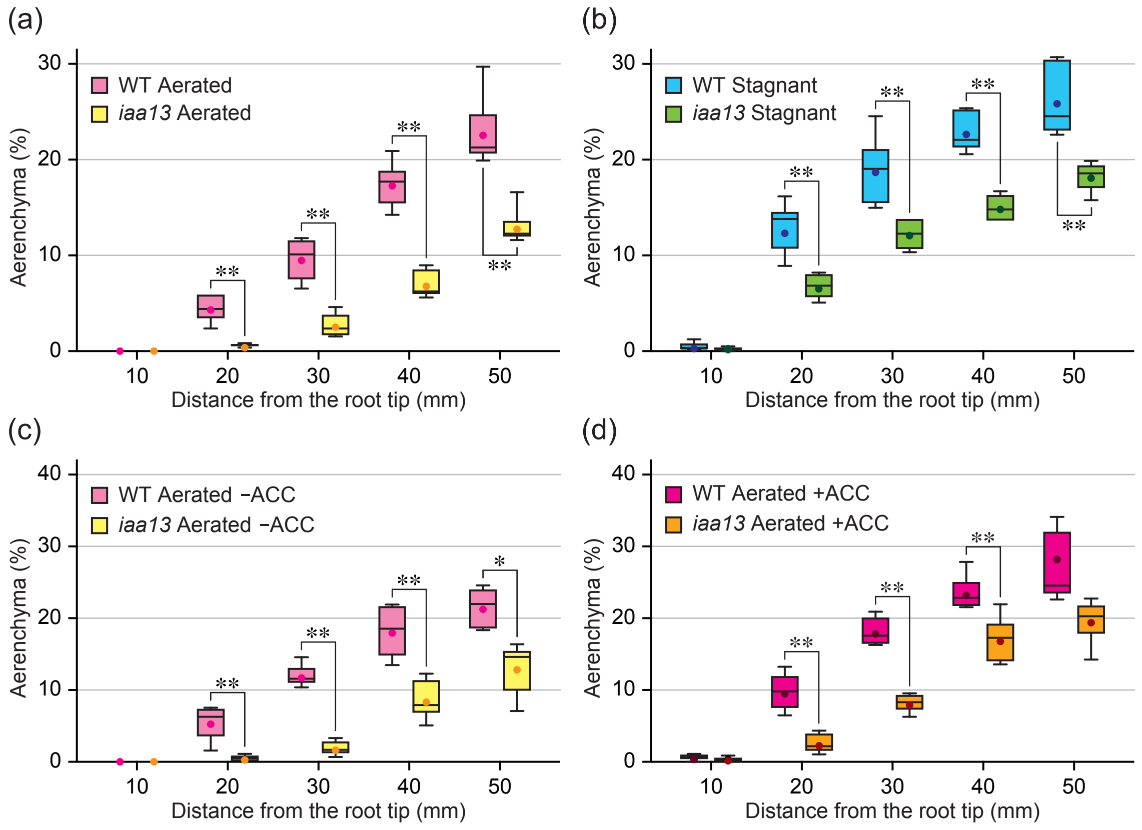

Supplemental Figure S2. Aerenchyma formation under aerated or stagnant conditions or aerated conditions with or without 1-aminocyclopropane-1-carboxylic acid (ACC) treatment. Percentages of aerenchyma in root cross-sectional area at 10, 20, 30, 40 and 50 mm.Twenty-day-old aerobically grown wild type (WT) and *iaa13* seedlings were further grown under aerated conditions (**a**) or stagnant conditions (**b**) for 48 h, or under aerated conditions with 10 μM ACC treatment (**c**) or without ACC treatment (**d**) for 48 h. Significant differences between the genotypes at $P < 0.05$ and $P < 0.01$ are denoted by * and **, respectively (two-sample *t* test). Boxplots show the median (horizontal lines), 25th to 75th percentiles (edges of the boxes), minimum to maximum (edges of the whiskers) and mean values (dots in the boxes) (n = 6).

Supplemental Table S1. List of primers used for qRT-PCR analysis

Gene name	ID (RAP-DB) [a]	ID (MSU) [b]	Forward primer sequence	Reverse primer sequence
ACS1	Os03g0727600	LOC_Os03g51740	5'-ACAAAACCACACCATGTCCA-3'	5'-CGAAAGGAATCTGCTACTGCTGC-3'
ACO5	Os05g0149400	LOC_Os05g05680	5'-CGAGTACCCGGAGTACGTGTT-3'	5'-ATTTTGGCGCCTTGACGGCC-3'
TFIIE	Os10g0397200	LOC_Os10g25770	5′-GTGCAGCCCAAGGCTAAG-3′	5′-CGTCGAATAAGCGTAGAGCA-3′

[a]RAP Os IDs in Rice Annotation Project Database (RAP-DB; http://rapdb.dna.affrc.go.jp/).

[b]MSU's Loc_Os IDs in Rice Genome Annotation Project Database (http://rice.plantbiology.msu.edu/).
